# Click-chemistry targeting of inflammation using NIR-emitting lanthanide nanoparticles with preserved optical functionality

**DOI:** 10.1039/d6ra06790e

**Published:** 2026-11-03

**Authors:** Livia Didonè, Dongmei Qiu, Paula Gutiérrez González, Marta Román-Carmena, Emma Martín Rodríguez, Jorge Rubio Retama, M. Carmen Iglesias-de la Cruz, Dirk H. Ortgies, Daniel Jaque, Miriam Granado, Álvaro Artiga

**Affiliations:** a Nanomaterials for BioImaging Group (nanoBIG), Faculty of Medicine, Department of Physiology, Universidad Autónoma de Madrid 28029 Madrid Spain miriam.granado@uam.es; b Department of Chemistry in Pharmaceutical Sciences, Complutense University of Madrid E-28040 Madrid Spain; c Nanomaterials for BioImaging Group (nanoBIG), Faculty of Science, Department of Materials Physics, Universidad Autónoma de Madrid 28049 Madrid Spain daniel.jaque@uam.es alvaro.artiga@uam.es; d Nanomaterials for BioImaging Group (nanoBIG), Faculty of Science, Department of Applied Physics, Universidad Autónoma de Madrid 28049 Madrid Spain; e Institute for Advanced Research in Chemical Sciences (IAdChem), Universidad Autónoma de Madrid 28049 Madrid Spain; f Instituto de Ciencia de Materiales Nicolás Cabrera, Universidad Autónoma de Madrid 28049 Madrid Spain; g Nanomaterials for BioImaging Group, Instituto Ramón y Cajal de, Investigación Sanitaria IRYCIS Ctra de Colmenar km 9300 28034 Madrid Spain

## Abstract

Bioorthogonal click chemistry was employed for the selective detection of VCAM-1 overexpression on inflamed endothelial cells using lanthanide-doped nanoparticles as near-infrared (NIR) probes. Neodymium/Ytterbium co-doped nanoparticles exhibited efficient and specific targeting following bioorthogonal conjugation, while preserving their characteristic NIR emission profile and thermal sensing capabilities. These results demonstrate the potential of this strategy for the selective recognition of inflammatory biomarkers without compromising the optical performance of the nanoprobes, paving the way for multifunctional platforms for inflammation imaging and nanothermometry.

## Abstract 2

Bioorthogonal click chemistry enables selective detection of VCAM-1 overexpression in endothelial cells using luminescent nanothermometers.

## Graphical abstract

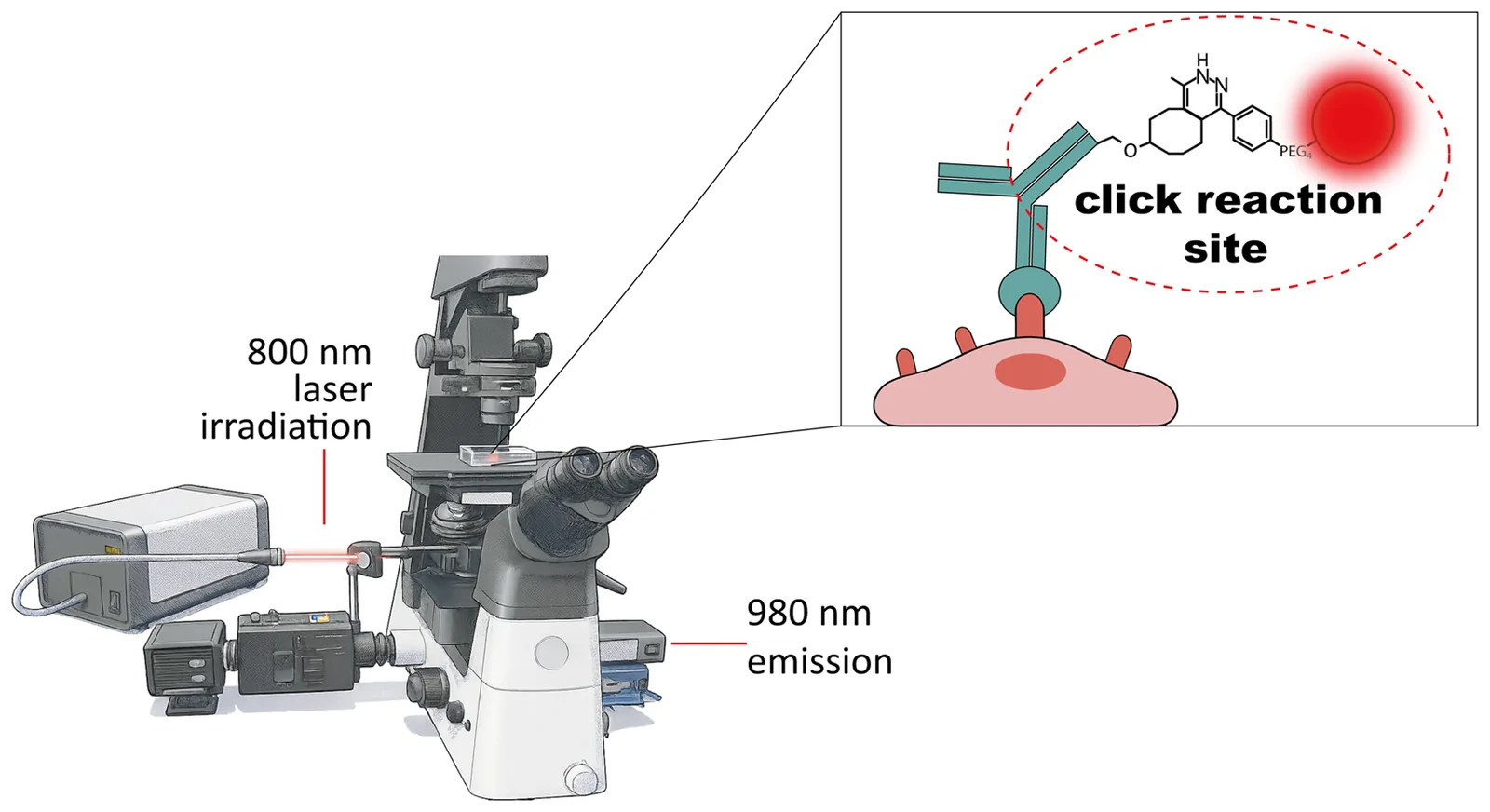

## Introduction

Inflammation plays a crucial role in many diseases, making the quest for sensitive and non-invasive detection probes of crucial interest for biomedical research and clinical diagnostics.^1,2^ Luminescent probes working in the near-infrared second biological window (NIR-II, 950–1450 nm) are particularly attractive as tissues become partially transparent in this spectral region enabling deep tissue *in vivo* imaging without requiring ionizing radiation or expensive equipment.^3–6^ Lanthanide-doped nanoparticles are of special interest due their unique combination of properties (long lifetimes, narrow emission lines, high spectral stability) that allow their use for auto-fluorescence free *in vivo* imaging in both the spectral and time domains.^7^ Furthermore, some of the lanthanide-doped nanoparticles working in the NIR-II behave as thermal reporters, becoming lanthanide-based luminescent nanothermometers (Ln-LNThs).^8^ NIR-II Ln-LNThs make the advanced diagnosis of pathologies that cause temperature alterations, such as inflammation, possible. Indeed, NIR-II Ln-LNThs have been already postulated for early detection of joint inflammation.^9–11^

The potential application of NIR-II Ln-LNThs for detection and diagnosis of inflammation requires their efficient accumulation in inflamed cells so that their luminescence signal can be used to localize them whereas their thermal readouts can be used for diagnosis. Since classical surface functionalisation procedures lead to limited accumulation of nanoparticles (NPs) in inflamed tissues and cells, mainly due to the screening effect caused by the protein corona, researchers are continuously looking for alternatives that could lead to an improvement in the targeting efficiency of NPs against inflamed tissues. Among the different alternatives explored, bioorthogonal click chemistry has attracted considerable attention due to its high efficiency, selectivity, and compatibility with biological systems.^12–16^ As an quantitative experimental evidence, Haun *et al.* directly compared antibody-conjugated magneto-fluorescent nanoparticles with a two-step TCO/Tz bioorthogonal targeting strategy, producing substantially greater nanoparticle binding compared to directly antibody-conjugated nanoparticles, with up to an approximately 15-fold increase in nanoparticle binding to HER2 (trastuzumab), EpCAM and EGFR (cetuximab) on HER2-positive cells. This enhancement was attributed to the amplification enabled by the two-step architecture, in which the number of available reactive sites for subsequent nanoparticle capture is increased.^17^

The demonstrations of inflammation detection by NIR-II luminescent nanoparticles and biorthogonal click chemistry are very scarce,^2,18^ and there is a knowledge gap regarding its performance at *in vitro* (cellular) level and also concerns to which extent the performance of NIR-II Ln-LNThs (as optical probes and thermal reporters) are affected by the functionalization procedure.

In this study, we faced this knowledge gap by evaluating the potential detection of inflamed cells by using NIR-II Ln-LNThs and by exploring how their thermal sensing properties are affected once attached to inflamed cells. As the case of study of NIR-II Ln-LNThs, we used neodymium and ytterbium co-doped NaYF_4_ nanoparticles with a core–shell architecture. These Ln-LNThs have been successfully used as thermal reporters in different biological scenarios based on the quenching of the emission generated by ytterbium ions that leads to noticeable changes in both lifetime and NIR-II emitted intensity (more details about Ln-LNThs used in this work can be found in SI) and for *ex vivo* bioorthogonal imaging of ischemia.^2,19,20^ As an inflammation model, a culture of immortalized mouse aortic endothelial cells (IMAEC) (Fig. 1, step 1), which upregulate vascular cell adhesion molecule-1 (VCAM-1) in response to inflammatory stimuli (Fig. 1, step 2), was optimized. VCAM-1 is expressed at the membrane level of endothelial cells, and plays a critical role in leukocyte recruitment to inflamed tissues,^21^ and recent evidence suggests that VCAM-1 is associated with the progression of a variety of immunological disorders, including autoimmune diseases and cancer.^22^ For this reason, developing novel methodologies for VCAM-1 overexpression is crucial for subsequent diagnosis. The increased expression of VCAM-1, characterised in Fig. S1, SI, occured when the endothelial layer was exposed to inflammatory conditions, in this case as a result of a 4-hour incubation of the cells with lipopolysaccharide (LPS) at 1 µg mL^−1^. LPS is a component of the outer membrane of bacteria and possesses immune-activating properties; indeed, its use is well established for inducing inflammatory models by stimulating the production of inflammatory cytokines.^23,24^ After LPS-induced VCAM-1 overexpression, the antibody against VCAM-1 (α-VCAM) functionalised with the click group *trans*-cyclooctene (TCO) was added to the cell culture, enabling selectively binding of VCAM-1 and exposing the TCO moieties (Fig. 1, step 3). Then, Ln-LNThs functionalised with tetrazine (Tz) groups (Ln-LNThs-Tz) were administered (Fig. 1, step 4). A Tz click group conjugated to Ln-LNThs bound selectively to the TCOs on the α-VCAM antibody, thus recognising the inflamed cells (Fig. 1, step 5). The bioorthogonal click reaction between the Tz groups on the nanoparticles and the TCO moieties on the antibody proceeds through an Inverse Electron Demand Diels–Alder (iEDDA) cycloaddition between electron-deficient 1,2,4,5-tetrazine and a strained dienophile (TCO). Between the three major classes of bioorthogonal click reactions that have emerged, the iEDDA results particularly valuable for biomedical applications, since it proceeds with extraordinarily fast second-order rate constants (*k*_2_ up to 10^6^ M^−1^ s^−1^ for tetrazine–TCO pairs),^25^ enabling efficient bioconjugation even at low concentrations of reactants. This remarkable reactivity stems from the significant ring strain in TCO and the strong electron-withdrawing character of the tetrazine, which together lower the activation energy for the cycloaddition. The reaction proceeds rapidly at physiological temperature and pH without requiring catalysts, produces nitrogen gas as the only byproduct, and exhibits excellent selectivity in complex biological environments. Furthermore, the iEDDA reaction is compatible with a wide range of biomolecules, including antibodies, peptides, proteins, and nanoparticles, and can be performed using various tetrazine and dienophile derivatives with tuneable reactivity and stability profiles.^26,27^ Finally, upon illumination with near-infrared excitation at 800 nm, the luminescence signal generated by NIR-II Ln-LNThs enables optical detection of the inflamed cells (Fig. 1, step 6).

**Fig. 1:**
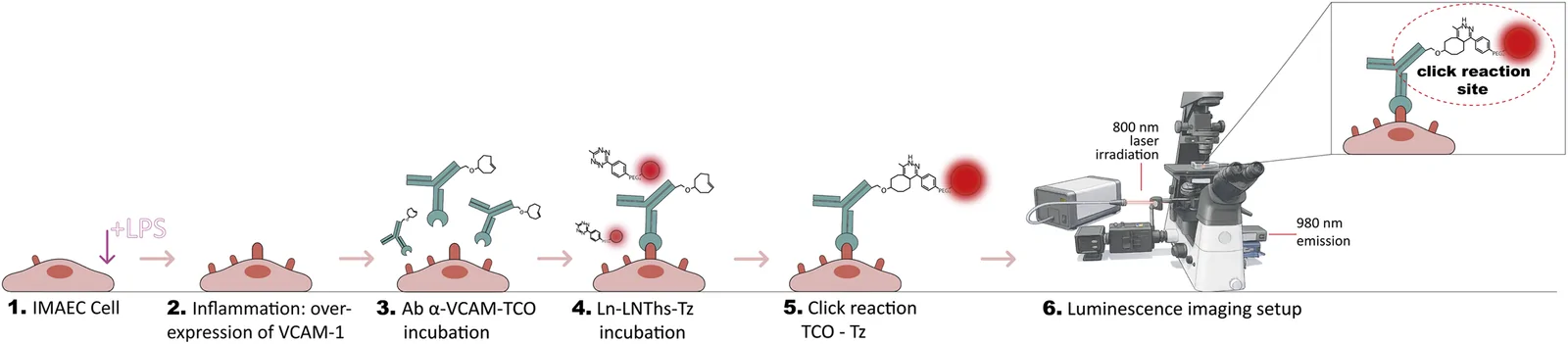
Experimental sequential scheme for VCAM-1 detection on inflamed IMAEC cells: (1) IMAEC cells were stimulated with LPS at 1 µg mL^−1^ for 4 hours, inducing (2) overexpression of VCAM-1; (3) incubation of α-VCAM-TCO antibodies for 1 hour; (4) subsequent incubation of Ln-LNThs-Tz for 30 min; (5) TCO-Tz click reaction binding the nanoparticles to the antibodies; (6) 800 nm laser excitation and NIR luminescent imaging to detect the site of the click reaction.

## Results and discussion

As a first step, we checked the functionality of the Ln-LNThs after their conjugation with tetrazine (Fig. 2). Ln-LNThs were synthesised and functionalised with the Tz moiety according to previous-described methodology.^2,28,29^ Ln-LNThs-Tz maintained their emission functionality, exhibiting identical photoluminescence profiles (Fig. 2b).

**Fig. 2:**
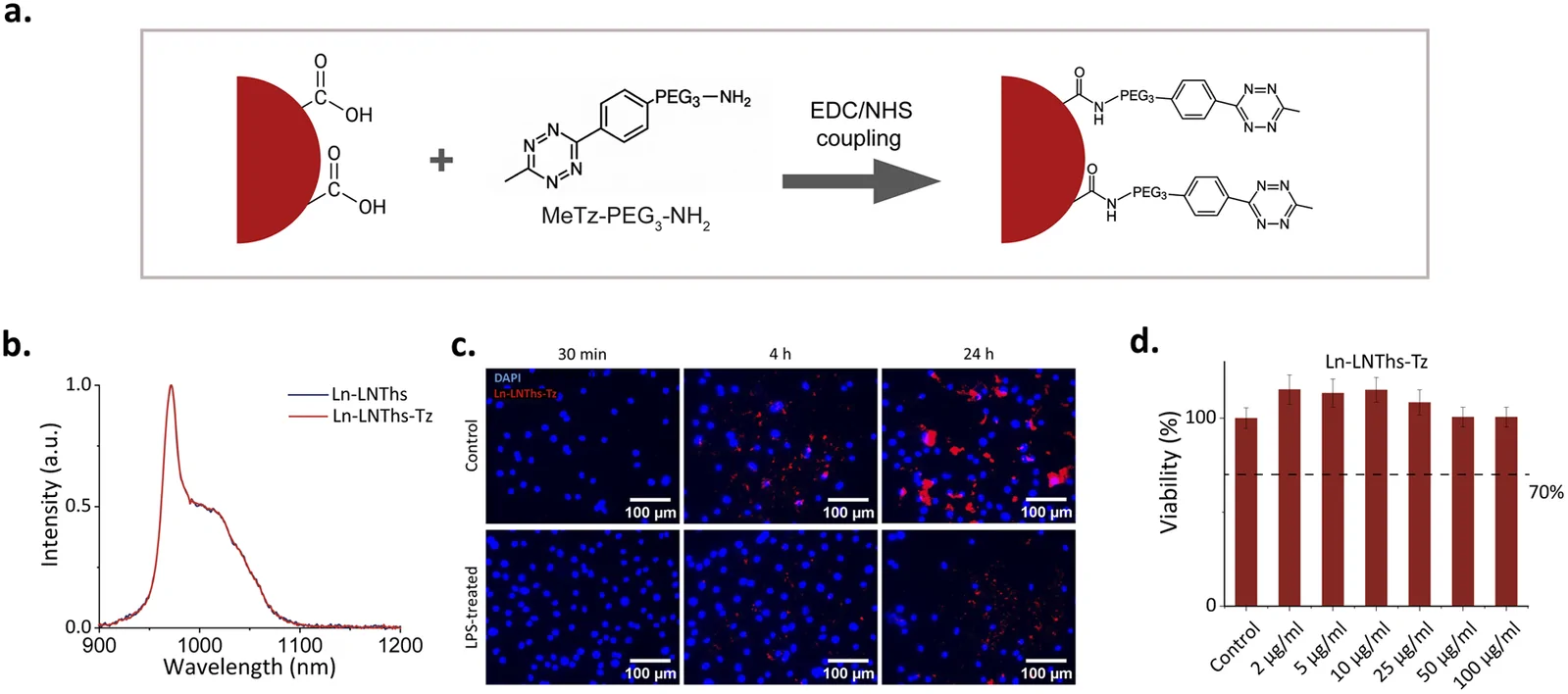
(a) Functionalization of Ln-LNThs covered with PAA (polyacrylic acid) with methyl-tetrazine (Me-Tz-PEG_3_-NH_2_) carried out by reaction between EDC-HCl and NHS coupling agents and the PAA-coated NPs. (b) Luminescent emission spectra of Ln-LNThs and Ln-LNThs-Tz, after excitation with 800 nm laser. (c) Fluorescence microscopy images of the internalisation study of 100 µg per mL Ln-LNThs-Tz after 30 minutes, 4 hours, and 24 hours of incubation in IMAEC cells in physiological (Control) and inflammation (LPS-treated) conditions, showing DAPI (blue) and NIR-II emission of Ln-LNThs (red) (d) MTT ((3-(4,5-dimethylthiazol-2-yl)-2,5-diphenyltetrazolium bromide)) cytotoxicity assay after 24-h incubation of IMAEC cells with different concentrations of Ln-LNThs-Tz nanoparticles. Cell viability is expressed as a percentage relative to untreated control cells and presented as mean ± SEM.

Another key experimental validation was to verify that Ln-LNThs-Tz were not internalized by the cells during incubation, thereby avoiding nonspecific signals and ensuring selective detection of VCAM-1 at the cell membrane in living cells under physiological (control) and inflammatory (LPS-treated) conditions. Cells were incubated with 100 µg per mL Ln-LNThs-Tz and assessed at early and prolonged time points (Fig. 2c). At the 30 min incubation time, no detectable nanoparticle-associated signal was observed in either control or inflamed cells, ruling out detectable early nanoparticle internalization or nonspecific nanoparticle adsorption under these experimental conditions. In contrast, nanoparticle-associated signals became evident after longer incubation times (4 h and 24 h), most likely reflecting their interaction with cell membrane and progressive cellular internalization. Therefore, the results support the interpretation that, during the 30 min click-labelling experiments, the nanoparticles are most likely interacting with membrane-associated VCAM-1 rather than being internalized, although exclusive plasma membrane localization cannot be directly demonstrated. Interestingly, prolonged incubation of Ln-LNThs-Tz resulted in lower nanoparticle-associated signals in LPS-treated cells compared with control cells, a trend that was already evident after 4 h and became more pronounced after 24 h. Although the purpose of this experiment was solely to verify the absence of significant nanoparticle internalization during the short incubation times employed in the pre-targeting protocol, these observations suggest that the inflammatory condition may influence nanoparticle uptake by endothelial cells. A possible explanation is that sustained LPS exposure induces cellular stress, damage, or death in a fraction of the cell population.^30^ Since nanoparticle uptake depends strongly on cellular viability and metabolic activity, inflammation-induced alterations in cell physiology could account for the reduced nanoparticle accumulation observed in LPS-treated cells after 24 h. This may be associated with the inflammatory and cellular stress response triggered by LPS. In agreement with this hypothesis, LPS stimulation increased nitrite/nitrate release in the culture medium, indicating enhanced nitric oxide production. Increased cellular stress may alter endocytic processes and/or intracellular nanoparticle retention, resulting in lower nanoparticle accumulation over prolonged incubation periods. Nevertheless, additional studies evaluating cell viability and specific stress-related pathways would be required to confirm this hypothesis. In addition, cytotoxicity studies of the Ln-LNThs-Tz indicated excellent biocompatibility establishing the safety profile of these functionalized nanoprobes for biological imaging applications in living cells (Fig. 2d).

As a preliminary step towards implementing the pretargeting strategy, intermediate studies were conducted to validate the individual components of the system and to ensure that antibody functionalisation did not compromise its biological activity and recognition capacity. These experiments aimed to find the adequate balance between maintaining the antigen recognition capacity of the antibody and ensuring sufficient TCO functionalisation to enable efficient click reactivity with the Ln-LNThs-Tz. Indeed, increasing the number of TCO moieties conjugated to the antibody is expected to improve the click reaction yield, but may simultaneously compromise antibody–antigen recognition as it has been described for antibody conjugation to nanoparticles.^31^ Therefore, the functionalisation level must be carefully optimized to reconcile both requirements.

Hence, in light of the inconsistencies in the literature regarding the number of TCO that can be conjugated, α-VCAM antibodies were functionalised with the click handle TCO at modification ratios of 1 : 10 and 1 : 100 of TCO moieties per each Ab molecule.^32,33^ The ability of αVCAM-TCO to target VCAM-1 was first evaluated by immunofluorescence using an AlexaFluor-488 secondary Ab.^34^ The results shown in Fig. 3a indicate negligible fluorescence in the negative control, while a strong fluorescent signal corresponding to VCAM-1 expression was detected in cells treated with the native αVCAM antibody. Importantly, endothelial cells stimulated with LPS exhibited a higher fluorescence intensity compared to untreated control cells, confirming the expected upregulation of VCAM-1 under inflammatory conditions. Comparing the two functionalised antibodies, the Ab αVCAM-TCO 1 : 10 conjugate maintained a binding profile similar to that of the native Ab, whereas the Ab functionalised with higher TCO loading (1 : 100) showed a reduction both in the fluorescence signal and, more importantly, in the selectivity, suggesting that excessive TCO during functionalisation compromises VCAM-1 recognition (Fig. 3b). These results indicate that increasing TCO loading on the antibody impairs its ability to recognise the VCAM-1 antigen, likely due to steric hindrance or structural perturbations introduced during functionalisation in the recognition sites as reported previously.^35,36^

**Fig. 3:**
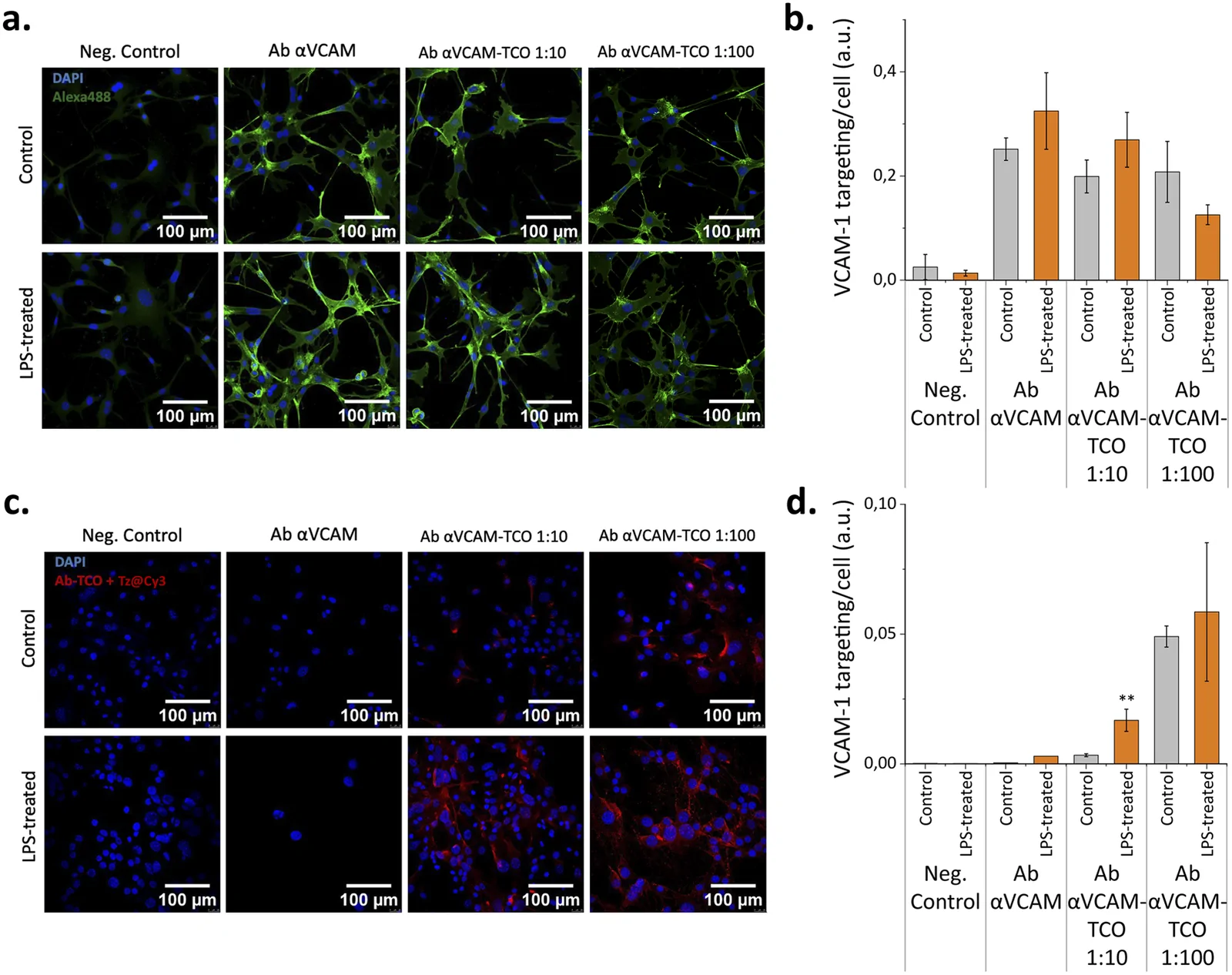
(a) Immunofluorescence assay to evaluate the targeting capability of click-functionalized anti-VCAM antibodies (Ab αVCAM) in IMAEC endothelial cells. Cells were stimulated with LPS for 4 h to induce inflammation and VCAM-1 overexpression, then fixed. Samples were incubated for 3 h with either native Ab αVCAM (control) or Ab αVCAM functionalized with different TCO ratios. After washing, cells were labelled with an Alexa-488 labelled secondary antibody (green) and counterstained with DAPI (blue) to visualise nuclei. (b) Quantification of Alexa-488 fluorescence intensity normalised to the number of cells per image. Data are presented as mean ± SEM. (c) Immunofluorescence assay evaluating click-mediated targeting using Ab αVCAM functionalized with different TCO ratios. Following LPS stimulation (4 h), and antibody incubation (1 h), cells were labelled with the Tz@Cy3 dye probe (red) and counterstained with DAPI (blue). (d) Quantification of Tz@Cy3 fluorescence intensity normalised to the number of cells per image. Results are reported as mean ± SEM, with statistical significance indicated as *p < 0.05 and **p < 0.005 compared to the control.

To confirm that the TCO groups linked to the Ab's surface remained chemically accessible and capable of undergoing the bioorthogonal reaction with Tz, an additional test was performed using a Tz-functionalised cyanine dye (Tz@Cy3) as a model click partner to evaluate the formation of Ab αVCAM-TCO-Tz conjugates. As shown in Fig. 3c, negligible fluorescence was detected with the non-functionalised αVCAM control, confirming the absence of nonspecific binding of the Tz@Cy3 to IMAEC cells. In contrast, cells treated with αVCAM-TCO exhibited a clear Cy3 fluorescence signal, demonstrating a successful bioorthogonal ligation between the TCO-modified antibody and the Tz-functionalised dye. The fluorescence intensity increased with higher TCO functionalisation, reflecting the greater number of reactive sites available for the click reaction. The quantitative analysis of this experiment, shown in Fig. 3d, further highlights the dependence of the click reaction efficiency on the degree of TCO functionalisation. However, while the αVCAM-TCO 1 : 100 conjugate produced the highest Cy3 signal due to its larger number of reactive TCO groups, this condition simultaneously showed reduced VCAM-1 selectivity compared with the previous targeting assay. In contrast, the αVCAM-TCO 1 : 10 conjugate provided a balanced and clear performance, maintaining strong antigen specificity while still enabling efficient click reactivity toward Tz-functionalised molecules.

Taken together, the results from these two complementary immunofluorescence assays demonstrate that the Ab αVCAM-TCO 1 : 10 functionalisation represents the most suitable functionalisation level among those evaluated, providing a favourable compromise between target recognition and click reactivity. This conjugation ratio preserves the antibody's ability to bind VCAM-1 selectively while ensuring sufficient availability of TCO groups to react with tetrazine-bearing probes. This observation is consistent with previous reports showing that moderate TCO labelling of antibodies better preserves antigen-binding affinity than extensive modification, which may compromise antibody recognition despite increasing the number of available click-reactive groups.^32,37,38^

Moreover, the stability of the αVCAM-TCO conjugate represents an important consideration for the future translation of this targeting strategy to *in vivo* applications. TCO moieties are known to progressively lose tetrazine reactivity in biological environments through *trans*-to-*cis* isomerization, with the extent of deactivation depending on the TCO structure and the molecular environment. Previous *in vivo* studies with TCO-modified monoclonal antibodies reported that approximately 75% of the TCO groups remained reactive 24 h after administration, suggesting a progressive rather than immediate loss of reactivity.^39^ Conversely, Rahim *et al.* observed comparable functional TCO levels on an anti-EGFR antibody immediately after conjugation and after 5 and 24 h at room temperature, indicating that TCO deactivation by *trans*-to-*cis* isomerization was negligible under those experimental conditions.^32^ Rather, they state that a lower functional reactivity of directly conjugated TCOs could happen predominantly due to hydrophobic masking within the antibody structure. These findings highlight that retained click reactivity depends not only on the chemical stability of the TCO moiety, but also on its accessibility and local molecular environment on the antibody surface.

Finally, the complete pretargeting strategy was evaluated using the αVCAM-TCO (1 : 10) conjugate in combination with Ln-LNThs-Tz probes. As shown in Fig. 4a, endothelial cells incubated in the presence/absence of LPS were first incubated with the αVCAM-TCO antibody to target the expression of VCAM-1 on the cell membrane. Afterwards, Ln-LNThs-Tz were added to trigger the bioorthogonal click reaction. Fluorescence microscopy images reveal minimal signal in the negative control, confirming the absence of nonspecific nanoparticle binding. In contrast, cells treated with αVCAM-TCO showed a clear luminescent signal from the Ln-LNThs-Tz probes, indicating successful click-mediated anchoring of the nanoparticles to the cell surface. Importantly, this signal is significantly enhanced in LPS-treated endothelial cells compared with untreated controls, consistent with VCAM-1 overexpression under inflammatory conditions.

**Fig. 4:**
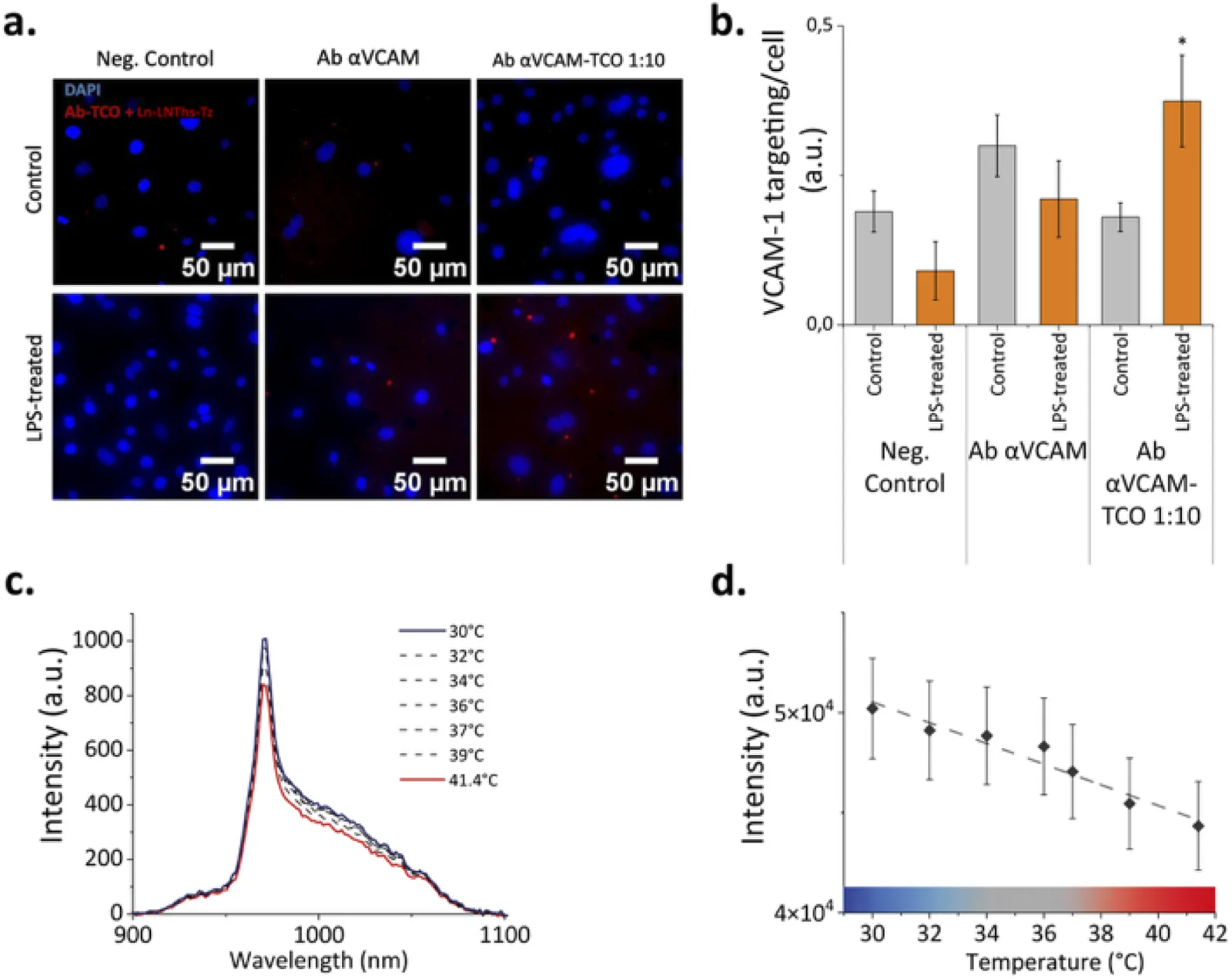
(a) Immunofluorescence assay following click chemistry labelling of IMAEC cells in physiological (control) and inflammation (LPS-treated) conditions, with Ab α-VCAM functionalized with TCO at a ratio 1 : 10 and Ln-LNThs-Tz. Cell nuclei are stained with DAPI (blue), while the Ln-LNThs NIR emission is in red. The negative control group was treated without any Ab; the Ab α-VCAM group was treated with the commercial antibody prior to any functionalization. (b) Quantification of the fluorescence signal shows the relative labelling efficiency under the different experimental conditions, reported as mean ± SEM. (c) Spectral emission profiles of the samples measured at different temperatures (30–41.4 °C) indicate temperature-dependent changes in the optical response while maintaining a consistent emission peak. (d) Summary of the relationship between temperature and the measured fluorescence signal, highlighting a progressive decrease with increasing temperature. Data are presented as mean ± SD, where the dashed line indicates the linear trend.

The quantitative analysis reported in Fig. 4b further confirms this observation, showing a marked increase in VCAM-1 targeting per cell in the LPS-stimulated group when the αVCAM-TCO antibody is used. This result demonstrates that the two-step pretargeting approach effectively translates the molecular recognition of VCAM-1 into selective accumulation of the luminescent nanoparticles at endothelial inflammation sites. The use of a bioorthogonal pretargeting strategy was deliberately selected over direct antibody–nanoparticle conjugation because it offers several advantages for targeted imaging applications. On one hand, this approach minimizes the need for direct attachment of bulky nanoparticles to the antibody, thereby reducing potential steric hindrance and preserving antigen-recognition capability. Indeed, our results show that increasing the degree of antibody modification negatively affects VCAM-1 recognition, highlighting the importance of limiting structural perturbations of the antibody. Furthermore, click-chemistry-based conjugation has been reported to provide more efficient and controlled bioconjugation than conventional carbodiimide-mediated approaches, preserving antibody functionality and orientation while improving target-binding performance.^40^ In addition, it has been demonstrated that copper-free click chemistry can substantially enhance antigen recognition and binding capacity compared with traditional EDC/NHS coupling methods.^41^ Finally, the pretargeting strategy allows the lanthanide nanoparticles to remain an independent and well-characterised platform, avoiding extensive nanoparticle–antibody coupling procedures that could potentially alter their colloidal, optical, or thermometric properties. Preservation of these optical and thermometric properties is further supported by the nanoparticle architecture, in which the thermosensitive Nd^3+^/Yb^3+^ layer is protected by an outer CaF_2_ shell that reduces surface-related quenching and limits interactions with the surrounding medium, thereby enhancing photoluminescence efficiency and thermometric performance.^29^ Moreover, from a translational perspective, the pretargeting strategy enables the use of a single, standardized nanoparticle formulation that can be adapted to different diseases simply by exchanging the targeting antibody. This modular approach may simplify manufacturing, quality control, and regulatory approval compared with producing a distinct antibody-functionalized nanoparticle formulation for each application.

Beyond validating the targeting capability of the pretargeting strategy, a key question is whether the thermal sensing properties of the Ln-LNThs are preserved once the nanoparticles are anchored to the membrane of inflamed cells. Since the thermometric performance of these nanoprobes relies on the temperature-dependent quenching of the ytterbium NIR-II emission, we systematically evaluated the quenching behaviour by recording emission intensity as a function of temperature both for Ln-LNThs-Tz in solution and for Ln-LNThs-Tz bound to LPS-stimulated endothelial cells *via* the bioorthogonal pretargeting approach. As shown in Fig. 4, the intensity-versus-temperature quenching curves obtained under both conditions are virtually identical, demonstrating that neither the chemical transformations involved in the bioorthogonal conjugation process nor the cellular microenvironment alter the thermal sensing response of the nanoprobes. This result confirms that the ability of Ln-LNThs to act as quantitative thermal reporters is fully retained after the complete pretargeting procedure, establishing their potential as nanothermometric tools for the diagnosis of inflammation at the cellular level.^42,43^

Overall, we demonstrate, for the first time, the detection of inflamed endothelial cells through VCAM-1 targeting using NIR-II emitting lanthanide nanoparticles combined with bioorthogonal click chemistry. We further show that neither the functionalisation process nor nanoparticle binding to inflamed cells induces appreciable changes in their luminescent properties, including emission spectra and thermal response. These results establish a reliable platform for cellular-level inflammation detection and suggest the potential of this approach for future thermal-based diagnosis of inflammatory processes.

## Experimental section

### Materials and methods

#### Synthesis of α-NaYF_4_ core Ln-LNThs

The cores were synthesised *via* the thermal decomposition of sodium and yttrium trifluoroacetates. First, 303.3 mg of YCl_3_·6H_2_O (1.0 mmol) was dissolved in 10 mL of trifluoroacetic acid (TFA) at 90 °C in a three-necked round-bottom flask. The mixture was then evaporated under a continuous nitrogen flow, resulting in Y(TFA)_3_ forming as a white powder. This solid was then dissolved in a mixture of oleic acid (OA, 3.2 mL), oleylamine (OLA, 3.2 mL), 1-octadecene (ODE, 6.4 mL) and sodium trifluoroacetate (NaTFA, 1 mmol, 136 mg). The solution was then heated to 110 °C, maintained at this temperature for 30 minutes under a vacuum, and then heated to 310 °C for a further 30 minutes. It was then allowed to cool naturally to room temperature under a nitrogen atmosphere. The resulting cores were harvested by centrifugation at 7000 g for 10 minutes after the addition of 20 mL of ethanol, washed three times with ethanol and finally dispersed in 10 mL of hexane for subsequent use.

#### Synthesis of α-NaYF_4_@NaYF_4_:Nd_0.6_Yb_0.2_ core@shell Ln-LNThs

Core@shell Ln-LNThs were prepared using a seed-mediated epitaxial growth procedure with the prepared α-NaYF_4_ as the core. To start with, the rare-earth trifluoroacetate (RE(TFA)_3_) shell precursors were synthesised using the same procedure as that reported above for the preparation of the Y(TFA)_3_ precursor, except that 0.2 mmol of YbCl_3_·6H_2_O, 0.6 mmol of NdCl_3_·6H_2_O and 0.2 mmol of YCl_3_·6H_2_O were used. Secondly, the RE(TFA)_3_ shell precursors were mixed with 10 mL of OA, 10 mL of ODE, 1.0 mmol (136 mg) of NaTFA and 0.5 mmol (≃90 mg) of the core Ln-LNThs in a three-neck flask. The mixture was then heated to 120 °C for 30 minutes under nitrogen gas and vacuum protection, then further heated to 300 °C for a further 30 minutes, before naturally cooling down to room temperature. The resulting core@shell Ln-LNThs were collected using the same procedure as for the α-NaYF_4_ cores and were dispersed in 10 mL of hexane for further use.

#### Synthesis of α-NaYF_4_@NaYF_4_:Nd_0.6_,Yb_0.2_@CaF_2_ core@shell@shell Ln-LNThs

The procedure for preparing the core@shell@shell rare earth (RE) nanoparticles (NPs) is similar to that for preparing the α-NaYF_4_@NaYF_4_:Nd_0.6_,Yb_0.2_ core@shell Ln-LNThs. For this, the previously synthesised α-NaYF_4_@NaYF_4_:Nd_0.6_,Yb_0.2_ core@shell Ln-LNThs were used as cores for seed-mediated growth of a CaF_2_ shell. Briefly, a mixture of 5 mL of a hexane dispersion of α-NaYF_4_@NaYF_4_:Nd_0.6_,Yb_0.2_ core@shell Ln-LNThs with a concentration of approximately 10 mg mL^−1^, 7 mL of OA and 7 mL of ODE was heated to 110 °C. This mixture was then maintained at this temperature under nitrogen gas and vacuum protection for 30 minutes. Subsequently, 3.2 mL of Ca(TFA)_2_ dissolved in OA (0.3 mmol mL^−1^) was injected into the solution in ten separate injections (0.32 mL each), with the solution being heated to 310 °C between each injection and then allowed to cool naturally to room temperature. The resulting core@shell@shell Ln-LNThs were then precipitated by centrifugation, washed twice with ethanol, and finally stored in 10 mL hexane.

#### Synthesis of clickable tetrazine-Ln-LNThs

In order to provide both aqueous stability and a surface to which the clickable group can be immobilised, the capping agents on the surface of the Ln-LNThs were first exchanged for poly(acrylic acid) (PAA). Typically, 5 mL of dimethylformamide (DMF) was added to 2 mL of the NPs in hexane solution (approximately 10 mg mL^−1^) and stirred for 10 minutes at room temperature. Then, 1.2 mmol (140 mg) of NOBF_4_ was dissolved in the solution of nanoparticles and the mixture was stirred vigorously for one hour at room temperature. Next, a toluene and hexane mixture (1 : 1 v/v) was added to flocculate the nanoparticles. After centrifugation at 10 000 g for 30 minutes, the NOBF_4_-capped NPs were dispersed in 2 mL of DMF to form a clear solution. Next, 500 mg of PAA was dissolved in 3 mL of DMF and mixed with the DMF dispersion of NOBF_4_-NPs. The reaction mixture was heated to 80 °C and kept under vigorous stirring overnight. The PAA-coated NPs were then precipitated with acetone, washed three times with ultrapure water, and finally dispersed in DMSO to form the stock solution.

#### Functionalization of Ln-LNThs with tetrazine

7 mg of lanthanide-doped nanoparticles coated with PAA (poly-acrylic acid) (Sigma-Aldrich, St. Louis, MO, USA) dispersed in 1 mL DMSO (Sigma-Aldrich, St. Louis, MO, USA) were activated with 1-ethyl-3-(3-dimethylaminopropyl)carbodiimide hydrochloride (EDC HCl, 2.0 mg; Sigma-Aldrich, St. Louis, MO, USA) and *N*-hydroxysuccinimide (NHS, 2.5 mg, Sigma-Aldrich, St. Louis, MO, USA) as coupling agents and allowed to react under agitation. This carbodiimide-mediated coupling strategy promotes the formation of stable amide bonds between the activated carboxylate groups on the nanoparticle surface and the primary amine functionality of the bifunctional linker. Subsequently, an aqueous solution of amino-functionalized methyltetrazine (H_2_N-PEG_3_-Me-Tz; 2.5 mg; Broadpharm, San Diego, CA, USA) was added to the activated nanoparticle suspension. The reaction was carried out under mildly basic conditions, achieved by the addition of *N*,*N*-diisopropylethylamine (DIPEA; 4 µL; Sigma-Aldrich, St. Louis, MO, USA), necessary to deprotonate the amine group and facilitate nucleophilic attack on the NHS ester intermediate. The resulting methyltetrazine-conjugated lanthanide-doped nanoparticles (Ln-LNThs-Tz) were recovered by centrifugation at 10 000 g for 30 minutes at room temperature, washed three times with 1 mL of DMSO to remove unreacted reagents and coupling agent by-products, and finally redispersed in 1 mL of DMSO (Sigma-Aldrich, St. Louis, MO, USA) for use.

#### Functionalization of Ab αVCAM with *trans*-cyclooctene (TCO)

Anti-vascular cell adhesion molecule-1 antibody (αVCAM-1; InVivoMab anti-mouse CD106, clone MK-2, IgG; Bio X Cell, catalog no. BE0027, Lebanon, NH, USA), was conjugated with *trans*-cyclooctene (TCO). Prior to conjugation, the antibody (200 µg) was buffer-exchanged into phosphate-buffered saline supplemented with 0.1 M sodium bicarbonate (NaHCO_3_) (final pH 8.5), using centrifugal ultrafiltration through 10 kDa molecular weight cut-off (MWCO) Amicon Ultra filters (UFC5010, MilliporeSigma, St. Louis, MO, USA) at 14 000 g for 10 minutes at 4 °C. Mildly alkaline pH promotes selective acylation of primary amine groups (lysine residues and the N-terminus) while minimizing hydrolysis of the activated ester. NHS-PEG_4_-TCO (Broadpharm, San Diego, CA, USA) was dissolved in anhydrous DMSO and added to the antibody solution, at the desired antibody-to-TCO molar ratio. The conjugation reaction was allowed to proceed overnight at 4 °C under mild shaking to ensure complete labelling while preserving antibody integrity and antigen-binding capacity. Following conjugation, unreacted NHS-PEG_4_-TCO and reaction by-products were removed by three successive washing and centrifugation cycles using 10 kDa MWCO Amicon® Ultra filters against PBS (pH 7.4) at 14 000 g for 10 minutes at 4 °C. The purified αVCAM-1-TCO conjugate was finally recovered by reverse centrifugation at 1 000 g for 3 minutes at 4 °C and stored at 4 °C until further use.

#### Immortalised murine aortic endothelial (IMAEC) cell culture

Immortalised murine aortic endothelial cells (IMAECs) were cultured in Dulbecco's Modified Eagle Medium/Nutrient Mixture F-12 (DMEM/F-12; Gibco, Thermo Fisher Scientific, Waltham, MA, USA) supplemented with 1% penicillin/streptomycin (Corning, NY, USA), 4 mM l-glutamine (Corning, NY, USA), 20% fetal bovine serum (Corning, NY, USA). The medium was further supplemented with endothelial cell growth factors (C-30120, PromoCell GmbH, Heidelberg, Germany). Cells were maintained under standard cell culture conditions (37 °C, 5% CO_2_). To support endothelial cell adhesion and monolayer formation, all culture surfaces were pre-coated with gelatin prior to cell seeding. Briefly, culture vessels were covered with a sterile aqueous solution of 0.5% gelatin (G1393, Sigma-Aldrich, St. Louis, MO, USA) and incubated at 37 °C for 2 hours. Following incubation, excess gelatin solution was aspirated, and the coated surfaces were used for cell seeding.

#### Cell viability assay

The cytocompatibility of Ln-LNThs and Ln-LNThs-Tz was evaluated using the MTT colourimetric assay, which provides an indirect measurement of cell viability, proliferation, and mitochondrial metabolic activity. The assay highlighted the metabolic activity of the cells thanks to the reduction (NADH/H+) from MTT (3-(4,5-dimethylthiazol-2-yl)-2,5diphenyltetrazolium bromide) (Fisher Scientific, Fair Lawn, NJ, USA), a yellow compound, to purple formazan crystals. These redox processes are directly proportional to the number of viable cells and can be quantitatively measured using an absorbance plate reader at 570 nm wavelength. IMAEC cells were seeded in gelatine-coated 96-well plates and exposed to serial dilutions of Ln-LNThs and Ln-LNThs-Tz over a concentration range of 2–100 µg mL^−1^ for 24 hours at 37 °C. Following incubation, culture medium was aspirated and replaced with fresh serum-free medium containing MTT solution 0.5 mg mL^−1^ and incubated for 4 h at 37 °C protected from light. The resulting formazan crystals were solubilised by addition of DMSO, and absorbance was measured at 570 nm. Untreated cells incubated with culture medium alone served as the negative control (100% viability). Cell viability was expressed as a percentage relative to the untreated control group and presented as mean ± standard error of the mean (SEM) from a minimum of *n* = 2 independent experiments, each performed in 8 technical triplicates. Cytocompatibility was assessed according to the ISO 10993-5:2009 standard for biological evaluation of medical devices, which defines a material as non-cytotoxic when cell viability at the highest tested concentration remains at or above 70% relative to the untreated control. Results are presented in Fig. S2, SI.

#### VCAM-1 overexpression quantification through qPCR

To assess mRNA expression and subcellular localization by qPCR, IMAEC cells were seeded on 6-well plates (3.5 × 10^5^ cells per well) and cultured to confluence (24–48 h). To induce endothelial inflammatory activation, cells were stimulated with lipopolysaccharide (LPS, 1000 ng mL; Sigma-Aldrich, St. Louis, MO, USA) in serum-free DMEM for 4 h at 37 °C and then washed with PBS. Meanwhile, controls cells were incubated with serum-free DMEM for the same amount of time. At the end of the incubation time, 1 mL of TRIzol Reagent (Invitrogen, Thermo Fisher Scientific, Waltham, MA, USA) was added directly to each well. Cells were scraped thoroughly using a cell scraper to ensure complete detachment and lysis, and the resulting lysate was transferred to a microcentrifuge tube. Samples were pipetted vigorously to ensure complete homogenization and incubated at room temperature for 5 minutes to allow complete dissociation of nucleoprotein complexes. For phase separation, 200 µL of chloroform (Sigma-Aldrich, St. Louis, MO, USA) was added per 1 mL of TRIzol, the tube was capped securely and shaken vigorously by hand for 15 seconds and incubated at RT for 15 minutes. Samples were then centrifuged at 12 000 g for 15 minutes at 4 °C. The upper aqueous phase, containing total RNA, was carefully transferred to a new tube, avoiding disturbance of the interphase and organic phase. RNA was precipitated by addition of 500 µL of isopropanol (Sigma-Aldrich, St. Louis, MO, USA), mixed gently by inversion, and incubated at room temperature for 10 minutes. Samples were centrifuged at 12 000 g for 10 minutes at 4 °C, and the resulting RNA pellet was washed with 1 mL of 75% ethanol by vortexing briefly and centrifuging at 7 500 g for 5 minutes at 4 °C. The ethanol was carefully aspirated, and the pellet was air-dried for 5–10 minutes at room temperature. The RNA pellet was resuspended in 20 µL of water. RNA concentration and purity were assessed spectrophotometrically using a NanoDrop spectrophotometer (Thermo Fisher Scientific, Waltham, MA, USA). Subsequently, 1 µg of total RNA was reverse-transcribed into complementary DNA (cDNA) using the High-Capacity cDNA Reverse Transcription Kit (Applied Biosystems, Foster City, CA, USA). Quantitative real-time PCR (RT-qPCR) was performed using TaqMan Gene Expression Assays (Applied Biosystems, Foster City, CA, USA) for VCAM-1 and the housekeeping gene hypoxanthine phosphoribosyltransferase 1 (Hprt1), using TaqMan Universal PCR Master Mix (Applied Biosystems, Foster City, CA, USA). Amplification was carried out on a StepOne Real-Time PCR System (Applied Biosystems, Foster City, CA, USA). Relative mRNA expression was calculated using the 2^−ΔΔ*C*_*t*_^ method,^44^ with cycle threshold (*C*_*t*_) values normalized to Hprt1 as the reference gene. Results were expressed as fold change relative to the unstimulated control group, which was set to 1 (or 100%).

#### VCAM-1 overexpression quantification through immunofluorescence

To assess protein expression and subcellular localization by immunofluorescence, IMAEC cells were seeded on glass coverslips in 24-well plates (0.5 × 10^5^ cells per well) and cultured to confluence (24–48 h). To induce endothelial inflammatory activation, cells were stimulated with lipopolysaccharide (LPS, 1000 ng mL; Sigma-Aldrich, St. Louis, MO, USA) in serum-free DMEM for 4 h at 37 °C and then washed with PBS. Meanwhile, controls cells were incubated with serum-free DMEM for the same amount of time. Cells were fixed with paraformaldehyde 2% for 10 min at room temperature (RT), and then with paraformaldehyde 4% for 10 min at RT, followed by two additional PBS washes to remove excess fixative. Non-specific binding sites were blocked by incubation with PBS containing 1% bovine serum albumin (BSA) for 30 minutes at RT. Cells were then incubated with primary antibodies for 3 h at RT: αVCAM-1 (InVivoMab anti-mouse CD106, clone MK-2, IgG; Bio X Cell, catalog no. BE0027, Lebanon, NH, USA) diluted 1 : 500 in PBS 1% BSA for non-click controls; or αVCAM-1-TCO diluted 1 : 100 in PBS 1% BSA. After washing three times with PBS 0.1% BSA, cells were incubated with Alexa Fluor 488 secondary antibody anti-mouse (dil. 1 : 1000; Abcam, Cambridge, UK) for 45 min, counterstained with DAPI (1 : 1000; Fisher Scientific, Fair Lawn, NJ, USA), mounted with VECTASHIELD antifade mounting medium (Vector Laboratories, Newark, CA, USA), and stored at 4 °C protected from light prior to imaging. Images were acquired using a Leica TCS SP5 confocal laser scanning microscope (Leica Microsystems, Wetzlar, Germany).

#### Immunofluorescence assay with Tz@Cy3 or Ln-LNThs-Tz

For live-cell click chemistry experiments, cells were seeded in 8-well IBIDI plates (0.3 × 10^5^ cells per well, 300 µL per well) and cultured to confluence (24–48 h). To induce endothelial inflammatory activation, cells were stimulated with lipopolysaccharide (LPS, 1000 ng mL; Sigma-Aldrich, St. Louis, MO, USA) in serum-free DMEM for 4 h at 37 °C and then washed with PBS. Meanwhile, controls cells were incubated with serum-free DMEM for the same amount of time. Cells were washed three times with PBS containing 0.1% BSA, then were incubated for 1 h at 37 °C with TCO-functionalized or non-functionalized primary antibodies diluted in serum-free DMEM supplemented with 1% penicillin/streptomycin and 1% BSA. After washing, the bioorthogonal click reaction was performed by incubating cells with tetrazine-functionalized probes (Tz-Cy3, 1 : 1000 from BroadPharm, San Diego, CA, USA), or (Ln-LNThs-Tz, 33 µg mL^−1^) for 30 min at 37 °C, followed by washing with PBS 0.1% BSA. Subsequently, cells were fixed with paraformaldehyde 2% for 10 min at room temperature (RT), and then with paraformaldehyde 4% for 10 min at RT, followed by two additional PBS washes to remove excess fixative. After washing, cells counterstained with DAPI (1 : 1000) (Fisher Scientific, Fair Lawn, NJ, USA), washed and stored in PBS at 4 °C until imaging. For the Tz@Cy3 immunofluorescence assay, fluorescence images were acquired using a Leica TCS SP5 confocal laser scanning microscope (Leica Microsystems, Wetzlar, Germany), with Cy3 fluorescence detected using the 543 nm laser line and emission collected in the 555–620 nm range. For immunofluorescence experiments involving Ln-LNThs-Tz, imaging of Ln-LNThs labelled samples was performed by infrared microscopy, as described in the following section.

#### Internalisation assay

IMAEC cells were seeded in 24-well plates (0.5 × 10^5^ cells per well) and cultured to confluence (24–48 h). To simultaneously induce inflammatory activation and initiate nanoparticle exposure, LPS (1000 ng mL; Sigma-Aldrich, St. Louis, MO, USA) and Ln-LNThs-Tz (100 µg mL^−1^) were co-administered in serum-free DMEM in a single treatment step. Cells were therefore incubated with the combined LPS/Ln-LNThs-Tz suspension in serum-free DMEM at 37 °C for 30 minutes, 4 hours, or 24 hours, to assess internalisation kinetics at early, intermediate, and late time points, respectively. Control cells were incubated with Ln-LNThs-Tz (100 µg mL^−1^) in serum-free DMEM alone, without LPS, for the same time points and under identical conditions, to evaluate nanoparticle uptake in the absence of inflammatory activation. At each designated time point, cells were washed twice with PBS to remove non-internalised nanoparticles and residual LPS. Cells were fixed with paraformaldehyde 2% for 10 min at room temperature (RT), and then with paraformaldehyde 4% for 10 min at RT, followed by two additional PBS washes to remove excess fixative. Following three PBS washes, cells were stored in PBS at 4 °C protected from light until imaging by infrared microscopy, as described in the following section.

#### Infrared microscopy

Near-infrared (NIR) fluorescence images were acquired using an adapted fluorescence microscopy setup based on a Nikon fluorescence microscope (Nikon, Tokyo, Japan). Excitation was provided by an 808 nm diode laser (FC-808, Changchun New Industries) coupled into the microscope optical path to illuminate the sample from bellow. The emitted NIR luminescence was collected through the microscope objective at the bottom and detected using an InGaAs NIR camera (C-RED2 First Light, Oxford Instruments). All NIR fluorescence images were acquired under identical excitation and camera acquisition settings to allow direct comparison of luminescence signals across different conditions.

#### Temperature-dependent spectral analysis

Following click chemistry labelling of the VCAM-1 overexpression with Ln-LNThs-Tz, IMAEC cells were detached from culture wells by trypsinisation (0.25%, Fisher Scientific, Fair Lawn, NJ, USA) for 5 minutes. Then DMEM was added to the cell suspension, which was collected and centrifuged at 255 RCF for 5 minutes to obtain a pellet. The supernatant was removed, and the cells were resuspended in 200 µL of PBS (Fisher Scientific, Fair Lawn, NJ, USA). The resuspended cells were transferred into an 8-well ibidi plate (ibidi, Gräfelfing, Germany) and allowed to rest for approximately 15 minutes to ensure that the cells settled onto the bottom of the wells. Fluorescence emission spectra were acquired using a spectrophotometer equipped for near-infrared (NIR) detection (NIRQuest, Ocean Insight, Orlando, FL, USA). Samples were loaded into an ibidi incubation chamber connected to the ibidi Temperature Controller – Silver Line to ensure precise and stable thermal conditions during the acquisition stage (ibidi, Gräfelfing, Germany). For quantitative analysis, fluorescence intensity values were extracted from the emission spectra at the peak wavelength (980 nm). The resulting intensity values were plotted as a function of temperature to assess thermal dependence. Data are presented as mean ± standard deviation (SD), obtained from independent measurements. A linear regression analysis was performed to evaluate the trend in fluorescence intensity as temperature increased.

## Conclusions

This work presents the first demonstration of VCAM-1-targeted detection of inflamed endothelial cells using NIR-II-emitting lanthanide nanoparticles in combination with bioorthogonal click chemistry. Importantly, we show that both nanoparticle functionalization and their selective binding to inflamed cells preserve the optical performance of the nanoplatform, including its luminescent emission and temperature-sensing capability. These findings establish a robust strategy for the selective detection of cellular inflammation and lay the foundation for future applications in NIR-II imaging and nanothermometry of inflammatory processes.

## Author contributions

Livia Didonè: writing – review & editing, writing – original draft, methodology, investigation, formal analysis, conceptualization. Dongmei Qiu: writing – review & editing, visualization, methodology, investigation. Paula Gutiérrez González: writing – review & editing, methodology, investigation. Marta Román-Carmena: writing – original draft, methodology, investigation. Emma Martín Rodríguez: writing – review & editing, methodology, investigation. Jorge Rubio-Retama: writing – review & editing, validation, supervision, resources, project administration, methodology, funding acquisition. María C. Iglesias-de la Cruz: writing – review & editing, validation, methodology, investigation, funding acquisition. Dirk H. Ortgies: writing – review & editing, writing – original draft, visualization, methodology, investigation, project administration. Daniel Jaque Garcia: writing – review & editing, writing – original draft, validation, supervision, resources, project administration, methodology, investigation, funding acquisition, conceptualization. Miriam Granado: writing – review & editing, writing – original draft, methodology, investigation, resources, formal analysis, validation, supervision, project administration, resources, funding acquisition, conceptualization. Álvaro Artiga: writing – review & editing, writing – original draft, visualization, supervision, methodology, investigation, formal analysis, validation, supervision, project administration, resources.

## Conflicts of interest

There are no conflicts to declare.

## Supplementary Material

RA-OLF-D6RA06790E-s001

## Data Availability

The data supporting the findings of this study are available from the authors upon request. Supplementary information (SI) is available. See DOI: https://doi.org/10.1039/d6ra06790e.
