# Supplementary material for: Click-chemistry targeting of inflammation using NIR-emitting lanthanide nanoparticles with preserved optical functionality
Source: RSC Adv. 2026 Nov 3. Online ahead of print. doi: 10.1039/d6ra06790e (PMC13730391; doi:10.1039/d6ra06790e)
Supplement: Supplementary file 1 [file RA-OLF-D6RA06790E-s001.pdf]

Supplementary Information (SI) for RSC Advances.
This journal is © The Royal Society of Chemistry

Supplementary Information

Click-Chemistry Targeting of Inflammation Using NIR-Emitting Lanthanide Nanoparticles with Preserved Optical Functionality

Livia Didonè,[a] Dongmei Qui,[b] Paula Gutiérrez González,[b] Marta Román-Carmena,[a] Emma Martín Rodríguez,[d,e,f,g] Jorge Rubio Retama,[b,g] María C. Iglesias-de la Cruz,[a,g] Dirk H. Ortgies,[c,e,f,g] Daniel Jaque,[c,e,f,g] Miriam Granado,[a,g] and Álvaro Artiga[c,g]

[a.] nanomaterials for BioImaging Group (nanoBIG), Faculty of Medicine, Department of Physiology, Universidad Autónoma de Madrid, 28029 Madrid, Spain

[b.] Department of Chemistry in Pharmaceutical Sciences, Complutense University of Madrid, E-28040 Madrid, Spain

[c.] nanomaterials for BioImaging Group (nanoBIG), Faculty of Science, Department of Materials Physics, Universidad Autónoma de Madrid, 28049 Madrid, Spain

[d.] nanomaterials for BioImaging Group (nanoBIG), Faculty of Science, Department of Applied Physics, Universidad Autónoma de Madrid, 28049 Madrid, Spain

[e.] Institute for Advanced Research in Chemical Sciences (IAdChem), Universidad Autónoma de Madrid, 28049 Madrid, Spain.

[f.] Instituto de Ciencia de Materiales Nicolás Cabrera, Universidad Autónoma de Madrid, 28049 Madrid, Spain.

[g.] nanomaterials for BioImaging Group, Instituto Ramón y Cajal de Investigación Sanitaria IRYCIS, Ctra de Colmenar km 9,300, 28034 Madrid, Spain.

SUPPLEMENTARY FIGURES

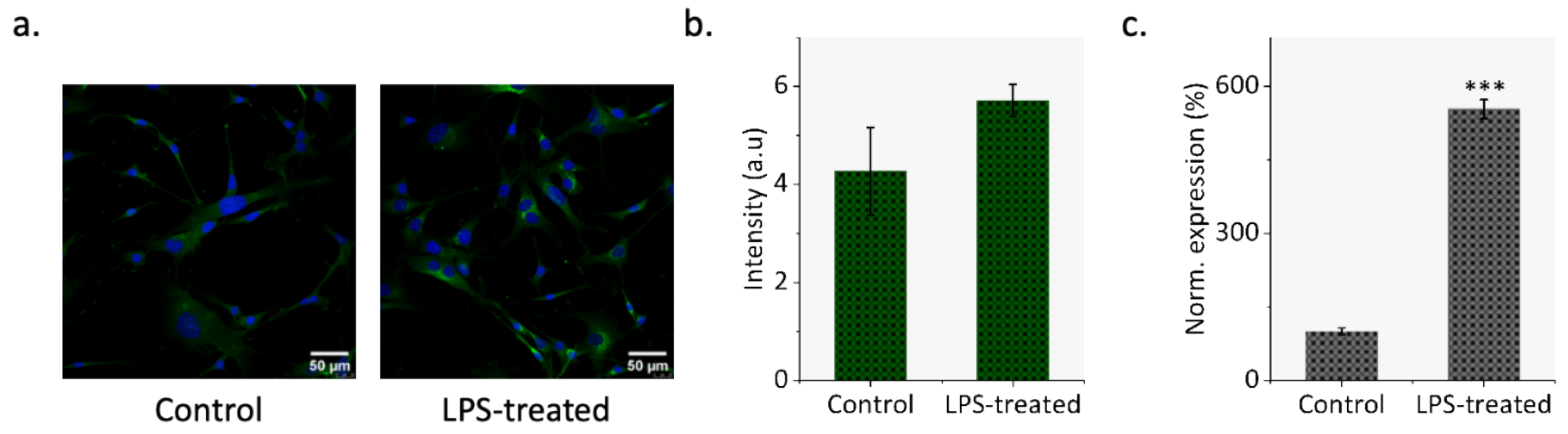

Figure S.1. a. Representative immunofluorescence images of VCAM expression in cells from expansion passage number 10. VCAM is shown in green and nuclei are stained with DAPI (blue). Scale bar = 50 μm. **b.** Quantification of VCAM immunofluorescence intensity in passage 10 cells showing elevated VCAM expression in the overexpression condition compared to control. Data are presented as mean ± SD. **c.** qPCR analysis confirming sustained VCAM mRNA overexpression in passage 10 cells relative to control samples. Data are presented as mean ± SD, and statistical significance is indicated (***p < 0.001).

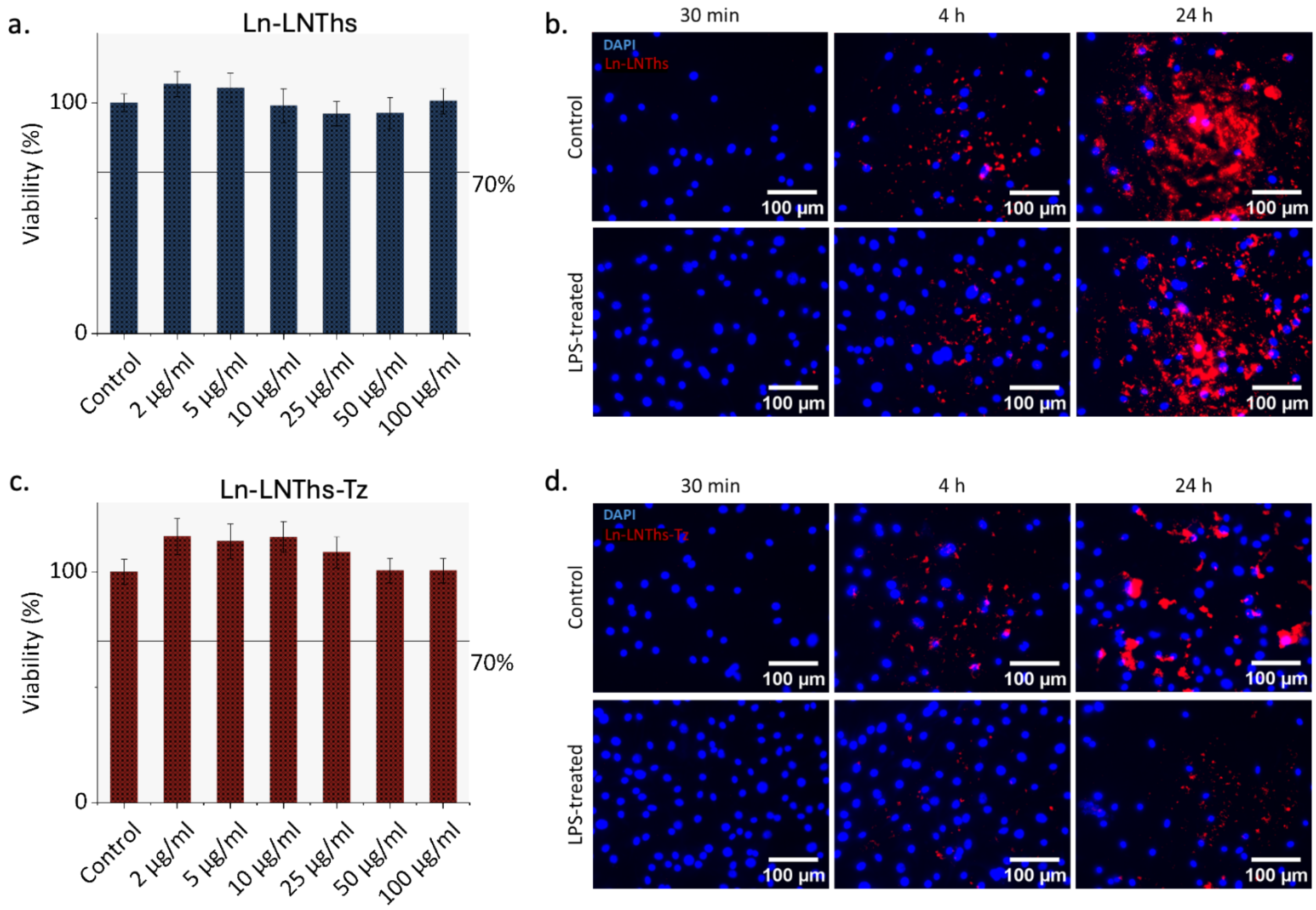

Figure S.2. a. and **c.** MTT ((3-(4,5-dimethylthiazol-2-yl)-2,5-diphenyltetrazolium bromide)) cytotoxicity assay after 24-h incubation of I-MAEC cells with Ln-LNThs nanoparticles and Ln-LNThs-Tz respectively. Cell viability is expressed as a percentage relative to untreated control cells and presented as mean ± SEM. According to the ISO 10993-5 acceptance criteria, materials are considered non-cytotoxic when the relative cell viability for the highest concentration of the test item extract is ≥ 70%. **b.** and **d.** Internalization study of 100 µg/ml Ln-LNThs and Ln-LNThs -Tz, respectively, after 30 minutes, 4 hours, and 24 hours of incubation in of I-MAEC cells in physiological (Control) and inflammation (LPS-treated) conditions.

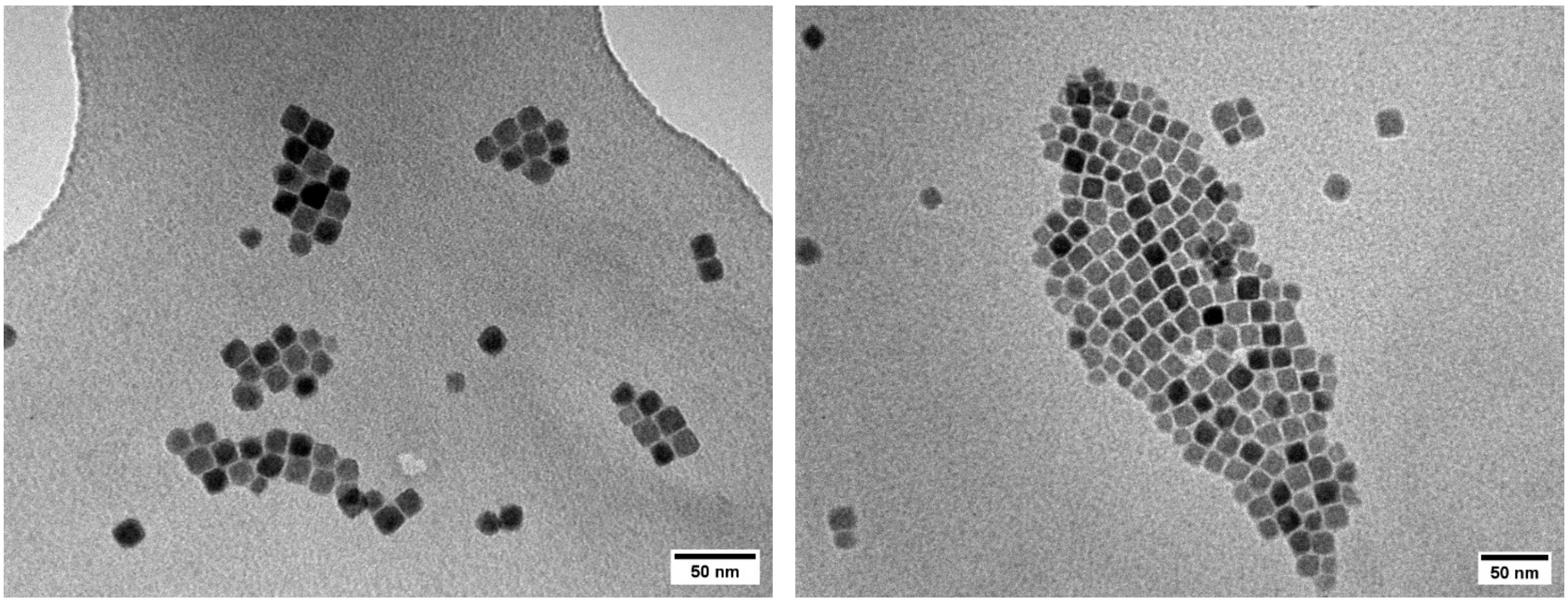

Figure S.3 Transmission electron microscopy (TEM) images of α-NaYF₄@NaYF₄:Nd$_{0.6}$,Yb₀$_{.2}$@CaF₂ core@shell@shell nanoparticles, showing a uniform size distribution with an average diameter of (16.99 ± 0.04) nm.

SUPPLEMENTARY COMPARISON WITH OTHER TARGETING STRATEGIES

To provide a broader comparison between the targeting strategies employed in this work and in previous studies, Table S1 summarizes the main characteristics of peptide-based active targeting and antibody-mediated bioorthogonal targeting, including their targeting mechanism, molecular requirements, reagent consumption, and associated economic considerations.

Parameter	Passive targeting	Active targeting	Bioorthogonal targeting
**Mechanism of action**	Relies on the EPR effect: NPs extravasation and retention[1]	Surface ligands recognise and bind specific receptors[2]	Two-step ligation[3]
**Targeting specificity**	None[1]	Dependent on receptor expression and accessibility[4]	High chemical selectivity: the bioorthogonal reaction only occurs between the click functional groups intentionally introduced on both the target and the NPs[5]

**Signal-to-background ratio**	Time-dependent: NPs gradually clear from healthy tissues and accumulate in tumour/pathological tissues[6]	Good at cellular level due to higher uptake.[4] 6–7× greater tumour accumulation[7]	Potentially high, due to separation of target recognition and probe administration and clearance of unbound components.[5] Can be up to 15× enhancement in nanoparticle binding[8]
**NPs surface modification**	Non required[6,2]	With the ligand[9]	With bioorthogonal functional groups[3]
**Tissue/tumour penetration**	Limited and heterogeneous[6]	Improved near vasculature[9]	Enables deep tumour penetration
**Pharmacokinetic**	Determined by NPs size and coating[2]	Targeting ligands can alter NP pharmacokinetics, potentially shortening circulation via increased opsonization; NP size and ligand type jointly modulate active targeting efficiency[9]	Targeting moiety and NP pharmacokinetics can be independently optimized; precise scheduling between pretargeting injection and NP injection is critical to maximize in vivo ligation. The higher halflife of the Ab can be exploited[5]

**Limitations**	Protein corona formation, RES clearance (liver/spleen), highly variable EPR across patients and tumour types; low intracellular uptake[2]	Off-target binding to receptor-expressing normal tissues; PEG/corona masking of ligands; heterogeneous receptor expression[9]	Multi-step administration complexity; challenges in efficient in vivo labelling; potential immunogenicity of non-native chemical groups; strict timing requirements; limited in vivo clinical experience[5]

Table S.1. Comparative overview of passive targeting, conventional active targeting, and bioorthogonal pretargeting strategies for nanoparticle-based molecular imaging. The comparison summarizes differences in targeting mechanism, specificity, signal-to-background characteristics, nanoparticle surface modification, tissue penetration, pharmacokinetic considerations, and major limitations.

Rather than requiring the preparation, purification, and optimisation of a distinct antibody–nanoparticle conjugate for every antibody/nanoparticle combination, the bioorthogonal approach decouples the targeting element from the nanoparticle platform. A common Tz-functionalized nanoparticle platform can therefore potentially be combined with different TCO-modified antibodies. We emphasise, however, that this modularity represents a potential advantage for platform development and manufacturing rather than an inherently lower cost per experiment, since the bioorthogonal strategy also requires TCO/Tz functionalization and sequential administration. These additions provide both quantitative experimental support and a more balanced discussion of the advantages and limitations of the pretargeting approach.

From an economic and translational perspective, the two targeting approaches investigated in previous studies from our group entail different sources of cost and operational complexity. As Muñoz-Ortiz *et al.* note,[10] in the conventional active-targeting strategy, nanoparticles are directly functionalized with the targeting antibody before administration, requiring antibody conjugation, purification, and quality-control steps for each nanoparticle formulation. By contrast, the Didonè *et al.* manuscript[11] states that the bioorthogonal approach separates molecular recognition from nanoparticle administration: the antibody is first functionalized with the bioorthogonal reactive group and administered as a pre-targeting agent, followed by injection of the complementary functionalized nanoparticles. Although this two-step strategy adds an administration step and may increase procedural costs, it may offer greater modularity, since the same bioorthogonally functionalized nanoparticle platform could, in principle, be combined with different targeting antibodies without redesigning and optimising a new antibody–nanoparticle conjugate for each molecular target. Based on the experimental workflows described in the two studies from our group, the main economic trade-off therefore lies between the simpler single-administration scheme of conventional active targeting and the potentially greater flexibility and reusability of a modular bioorthogonal platform.

Importantly, these considerations are a qualitative extrapolation from the experimental procedures and reagent requirements reported in the two studies, not a formal pharmacoeconomic analysis; therefore, dedicated translational and large-scale manufacturing studies would need to establish the actual cost-effectiveness of either strategy.

In the mentioned manuscripts, the substantially different molecular nature of peptide- and antibody-based targeting agents is considered when comparing their economic impact.

**Muñoz-Ortiz *et al.*,direct antibody conjugation**[10]	**Didonè *et al.*, bioorthogonal pretargeting strategy**[11]
In the cLABL-functionalized GNS system, approximately 3.3 μg of peptide are used during the functionalization of the amount of nanoparticles corresponding to 1 mL of experimental dispersion at 5 μg/mL. Based on a commercial price of €2006 for 11 mg of cLABL, this corresponds to an estimated peptide cost of approximately 0.60€. To be theoretically comparable with an antibody at equivalent molar amount, the 3.3 μg of peptide have a molecular weight of approx. 1.17 kDa, corresponding to 2.80 nmol. An equivalent molar amount of a 150-kDa IgG would require approximately 420 μg of antibody, corresponding to an estimated cost of approximately 402€.	In the bioorthogonal pretargeting strategy, 200 μL of antibody–TCO at 100 μg/mL are used per experiment, corresponding to: $200\ \square L \times \frac{100\ \square g}{mL} = 2$ μg antibody<br>The antibody costs 479 € for 500 μg,<br>corresponding to: $\frac{479\ €}{500\ \square g} = 0.958$ €/μg<br>Therefore: 2 μg × 0.958 €/μg = 1.92 €<br>The contribution of TCO to the overall reagent cost is negligible compared with that of the antibody, since the proportional rate of TCO used is 0,3419/200 × 20 = 0,03419 μg. Known that TCO's price is 287€/25.000 μg = 0,01148€/μg. Hence for 0,03419 μg: 0,03419 × 0,01148 ≈ 0,00039€
**Estimated antibody cost: 402€**	**Estimated antibody cost: 1.92€**

Table S2. Comparison of targeting-ligand requirements and estimated reagent costs for the cLABL peptide-based nanoparticle functionalization strategy reported by Muñoz-Ortiz *et al.* and the antibody-based bioorthogonal pretargeting strategy. The comparison considers the markedly different molecular weights and amounts of peptide and antibody required and is intended to illustrate reagent-cost and molecular-dose differences rather than provide a formal pharmacoeconomic assessment.

Together with the high specificity and modularity offered by click chemistry, this makes the bioorthogonal strategy an attractive compromise between targeting performance, reagent consumption, and platform adaptability.

Bibliography:

1 M. A. Subhan, S. S. K. Yalamarty, N. Filipczak, F. Parveen and V. P. Torchilin, *Journal of Personalized Medicine*, 2021, **11**, 571.

2 M. Wang and M. Thanou, *Pharmacological Research*, 2010, **62**, 90–99.

3 S. H. Lee, O. K. Park, J. Kim, K. Shin, C. G. Pack, K. Kim, G. Ko, N. Lee, S.-H. Kwon and T. Hyeon, *Journal American Chemical Society*, 2019, **141**, 13829–13840.

4 W. M. MacCuaig, B. L. Fouts, M. W. McNally, W. E. Grizzle, P. Chuong, A. Samykutty, P. Mukherjee, M. Li, J. B. Jasinski, B. Behkam and L. R. McNally, *ACS Appl. Mater. Interfaces*, 2021, **13**, 49614–49630.

5 L. Taiariol, C. Chaix, C. Farre and E. Moreau, *Chem. Rev.*, 2022, **122**, 340–384.

6 N. Bertrand, J. Wu, X. Xu, N. Kamaly and O. C. Farokhzad, *Adv Drug Deliv Rev*, 2014, **66**, 2–25.

7 Y. Xu, H. Wu, J. Huang, W. Qian, D. E. Martinson, B. Ji, Y. Li, Y. A. Wang, L. Yang and H. Mao, *Theranostics*, 2020, **10**, 2479–2494.

8 J. B. Haun, N. K. Devaraj, S. A. Hilderbrand, H. Lee and R. Weissleder, *Nat Nanotechnol*, 2010, **5**, 660–665.

9 T. D. Clemons, R. Singh, A. Sorolla, N. Chaudhari, A. Hubbard and K. S. Iyer, *Langmuir*, 2018, **34**, 15343–15349.

10 T. Muñoz-Ortiz, J. Hu, F. Sanz-Rodríguez, D. H. Ortgies, D. Jaque, D. Méndez-González, R. Aguilar, F. Alfonso, F. Rivero, E. Martín Rodríguez and J. García Solé, *Nanomedicine: Nanotechnology, Biology, and Medicine*, DOI:10.1016/j.nano.2022.102556.

11 L. Didonè, P. G. González, D. Qiu, G. Villaverde, Á. Artiga, D. H. Ortgies, M. Román-Carmena, S. Amor, Á. L. García-Villalón, M. Granado, D. J. Garcia and J. Rubio-Retama, *Colloids and Surfaces B: Biointerfaces*, 2026, **259**, 115332.
